# Large brain size is associated with low extra‐pair paternity across bird species

**DOI:** 10.1002/ece3.8087

**Published:** 2021-09-14

**Authors:** Min Chen, Guopan Li, Jinlong Liu, Shaobin Li

**Affiliations:** ^1^ College of Life Science Yangtze University Jingzhou China; ^2^ MOE Key Laboratory of Biodiversity and Ecology Engineering Beijing Normal University Beijing China

**Keywords:** brain size, extrapair paternity, mating system, parentage, phylogenetic comparative analysis

## Abstract

**Background:**

Gaining extrapair copulations (EPCs) is a complicated behavior process. The interaction between males and females to procure EPCs may be involved in brain function evolution and lead to a larger brain. Thus, we hypothesized that extrapair paternity (EPP) rate can be predicted by relative brain size in birds. Past work has implied that the EPP rate is associated with brain size, but empirical evidence is rare.

**Methods:**

We collated data from published references on EPP levels and brain size of 215 bird species to examine whether the evolution of EPP rate can be predicted by brain size using phylogenetically generalized least square (PGLS) models and phylogenetic path analyses.

**Results:**

We found that EPP rates (both the percentage EP offspring and percentage of broods with EP offspring) are negatively associated with relative brain size. We applied phylogenetic path analysis to test the causal relationship between relative brain size and EPP rate. Best‐supported models (ΔCICc < 2) suggested that large brain lead to reduced EPP rate, which failed to support the hypothesis that high rates of EPP cause the evolution of larger brains.

**Conclusion:**

This study indicates that pursuing EPCs may be a natural instinct in birds and the interaction between males and females for EPCs may lead to large brains, which in turn may restrict their EPC level for both sexes across bird species.

## Graphical abstract

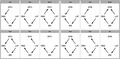

## 1 INTRODUCTION

Extra‐pair paternity (EPP) is prevalent in avian species (Cockburn, 2006). Based on an overview of recent literature reporting EPP in 255 socially monogamous avian species with biparental care, genetic polyandry has been detected in 76% of species, with great variation in the level across surveyed species (Brouwer & Griffith, 2019). A big question that remains unclear across species is why EPP is high in some species (e.g., Meliphagidae with 60% of offspring sired by extrapair male), whereas it is rare in others (e.g., carnivorous Laniidae; Brouwer & Griffith, 2019). There are a number of adaptive hypotheses proposed to explain EPP variations, such as fertility insurance hypothesis (Sheldon, 1994), genetic diversity hypothesis (Westneat et al., 1990), genetic compatibility hypothesis (Tregenza & Wedell, 2000), good gene hypothesis (Birkhead & Møller, 1992), and direct benefit hypothesis (Burke et al., 1989). However, past studies did not detect a general pattern across all avian species, though several different hypotheses have explained EPP rate variation at different extent in some species (Brouwer et al., 2017; Cockburn, 2004; Du & Lu, 2009). All those hypotheses proposed to explain EPP variation suggested procuring extrapair copulation (EPC) is a complicated and intelligent behavior, as a number of behavioral limitations (e.g., territorial behavior and mate guarding; West, 2014) exist during the process.

A complicated and intelligent behavior (e.g., procuring EPCs) is often associated with large brain capacity, which can increase their fitness (Allman et al., 1993; Lefebvre, 2013). Some hypotheses predict that both sexes attempt to outsmart each other to gain EPCs for increasing fitness in birds (Cockburn, 2004; West, 2014): Females attempt to sneak in EPCs in case of reduced male parental care; males attempt to prevent females' EPCs while they gain their own EPCs. This interaction between females and males could lead to an increase in brain size (relative to body mass). However, those hypotheses did not receive much empirical evidence, though some studies have found that EPP could be affected by relative brain size. For example, West (2014) found in principal component analyses that multiple selective regimes (including EPP and several life history traits) correlate with large brain size. However, this study was based on 42 species and EPP only accounted for 0.3% of the variance explained by components. Another comparative analysis based on data of 38 species revealed that species with high levels of EPP have larger‐brained females than males, whereas females in species with low levels of EPP have smaller brains than males (Garamszegi et al., 2005). This study suggested that EPP rate only shaped females' brain evolution, but their small samples likely biased the results.

A recent intensive comparative analysis does not find clear evidence that EPP variation across species can be explained by ecological or life history factors (e.g., breeding synchrony, density, migration, generation length, genetic structuring, or climatic variability; Brouwer & Griffith, 2019). EPP patterns across species remain puzzling (Brouwer & Griffith, 2019; Cockburn, 2004). Latitude is often used as a proxy of breeding synchrony in a number of studies to test its effect on EPP variation (Brouwer & Griffith, 2019; Spottiswoode & Møller, 2004). Latitude is also known to be linked with many ecological factors (e.g., breeding density, climate variability, and primary productivity) and life history traits (e.g., annual adult survival and migration; Cardillo, 2002; Gillman et al., 2015; Muñoz et al., 2018). Avian species show substantial interspecific variation in relative brain size (Sayol et al., 2016), which were inferred to be related to the occurrence of EPCs (Garamszegi et al., 2005; West, 2014). In pair‐bonding species, both sexes are expected to be under selection pressure for larger brains due to the process of engaging in EPCs (West, 2004), because the females attempt to sneak in EPCs and increase the EPP level in their own brood, while the males guard their mates and add to the EPP rates of other nests by engaging in EPCs. All these behaviors can contribute to high levels of EPP in the population since there is a significant positive association between the rates of extrapair copulation and extrapair paternity (Birkhead & Møller, 1995).

In this paper, we collected published data on relative brain size and EPP rate in birds. The causal relationship between relative brain sizes (mean values of both sexes) and EPP rate across species, to our knowledge, was not tested across species in previous studies. Smaller samples more likely reach biased results as mentioned above (Garamszegi et al., 2005; West, 2014). Strong phylogenetic bias may also exist in EPP rates, with species with similar EPP rates clustered in the phylogeny (Brouwer & Griffith, 2019). Thus, we conducted a phylogenetic generalized least square (PGLS) regression and phylogenetically informed path analyses to test whether relative brain size explained interspecific variation in EPP rate across avian species, while also controlling for effect of latitude as a proxy of breeding synchrony (also indirectly controlling for some other ecological factors and life history traits mentioned above), which is supposed to potentially affect the EPP across species (Spottiswoode & Møller, 2004). Acquiring EPCs is intelligent behavior, and both sexes attempt to outsmart each other to gain EPCs to increase their fitness. Species with such tactical behavior are supposed to evolve large brains (Benson‐Amram et al., 2016; van der Bijl et al., 2015; West, 2004). Therefore, we predicted that large brains would lead to high EPP rate in birds.

## 2 METHODS

Large datasets are necessary for phylogenetic comparative analysis; although data for brain size in bird species are available for a larger number of species, the main limitation was the availability of EPP data. We used whole brain size in our analyses because such data are widely available (Jiménez‐Ortega et al., 2020; Sayol et al., 2016, 2018). Brain size and body mass were collated from Dunning (2008) and Sayol et al. (2016, 2018). EPP data were taken from a recent intensive review, which reported the current 30‐year literature on EPP level in avian species (Brouwer & Griffith, 2019). These datasets provide both the percentage EP offspring (EPO) and percentage of broods with EPO (EPB).

For cooperatively breeding (CB) species with more than two adults providing care for a brood of offspring, the EPP rate is much more complicated than that of biparental species because more potential breeders are involved within breeding group and female promiscuity within breeding group is not equal to the EPP rate. Thus, we only extract biparental broods of CB species to estimate the EPP rate in this study. We collated both data of EPO and EPB from Brouwer and Griffith (2019). When EPO or EPB were reported from different populations of the same species, we used their weighted mean values for later analyses. We also compiled those data from newly published studies through Web of Knowledge and Google Scholar (using the keywords such as “paternity,” “parentage,” or “brain size” in combination with “bird”). Some recent studies reported EPP rate of a few avian species, but their brain size was not available from the literature. Therefore, we exclude those species. Only the species with both brain size and EPP rate (either EPO or EPB) available were included in the dataset. Finally, we collected the data from 215 species (206 species with data of EPO and 211 species with data of EPO; see Supplementary materials).

Latitude (as a proxy of breeding synchrony) was considered to affect the EPP rate in some species (Spottiswoode & Møller, 2004). This variable is also associated with many other factors, such as environmental factors such as climate seasonality and primary productivity, and life history traits, such as annual adult survival and migration (Cardillo, 2002; Gillman et al., 2015; Muñoz et al., 2018). Such environmental factors and life history traits are considered to potentially affect the EPP rate (Brouwer & Griffith, 2019; Cockburn, 2004), so we included absolute latitudes as a covariate to control for a few confounding effects. Coordinates were either obtained directly or estimated from Google Earth based on the descriptions of study sites when their geographic latitudes were not reported. When EPP or EPB with their geographic latitudes was reported in different populations of the same species, we used their mean absolute values in later analyses.

For most species (152 out of 215), there is only one population estimate (from a single study) for the rate of EPP at the offspring or brood level available, though it would be necessary to understand the extent to which a single measure represents a species well. For species (*n* = 64) that have been investigated in more than one population, there was strong and significant repeatability of EPP rate at the species level: EPO's *R* = 0.755 ± 0.036 and EPB's *R* = 0.666 ± 0.078; estimated with package rptR on GLMM with EPP rate (percentage EPO and EPB, respectively) fitted as a response with identity of species and population included as random intercepts (Stoffel et al., 2017). These results show that repeatability is high and more than 66% of the EPP variation among the species that have been sampled in multiple populations could be attributed to variation at the species level, with a smaller part (less than 34%) of this variation due to variation within populations. Therefore, a single measure to a large extent can represent a species in this study.

Data from these species may be nonindependent for statistical analysis since data of closely related species tend to be similar because of their shared phylogenetic history (Felsenstein, 1985; Harvey & Pagel, 1991). So, we applied phylogenetic generalized least squares (PGLS) approach to controlling for nonindependence of data. We downloaded 100 fully resolved trees from the Bird Tree project (Jetz et al., 2012) using the Hackett backbone (Hackett et al., 2008) for all our species. With the 100 trees, we built the maximum clade credibility tree (summary tree) using the package *phangorn* (Schliep, 2011) in R (R Core Team, 2018). Relative brain size was estimated as the residual of brain size against body size from a log–log PGLS regression through the summary tree (Revell, 2012). Furthermore, we conducted PGLS models to test whether EPP rate (percentage EPO and EPB as a response variable, separately) is associated with relative brain size, while including latitude as a covariable. We applied a maximum‐likelihood estimation of Pagel's *λ* for phylogenetic dependence. Phylogenetic dependence (*λ*) was tested against a value of 0 (the evolution of a trait is independent of phylogeny) and a value of 1 (complete phylogenetic dependence; Freckleton et al., 2002). Phylogenetic signal was considered to be present if *λ* differed significantly from 0 even if it differed statistically from 1 (Freckleton et al., 2002; Revell, 2010).

**FIGURE 1:**
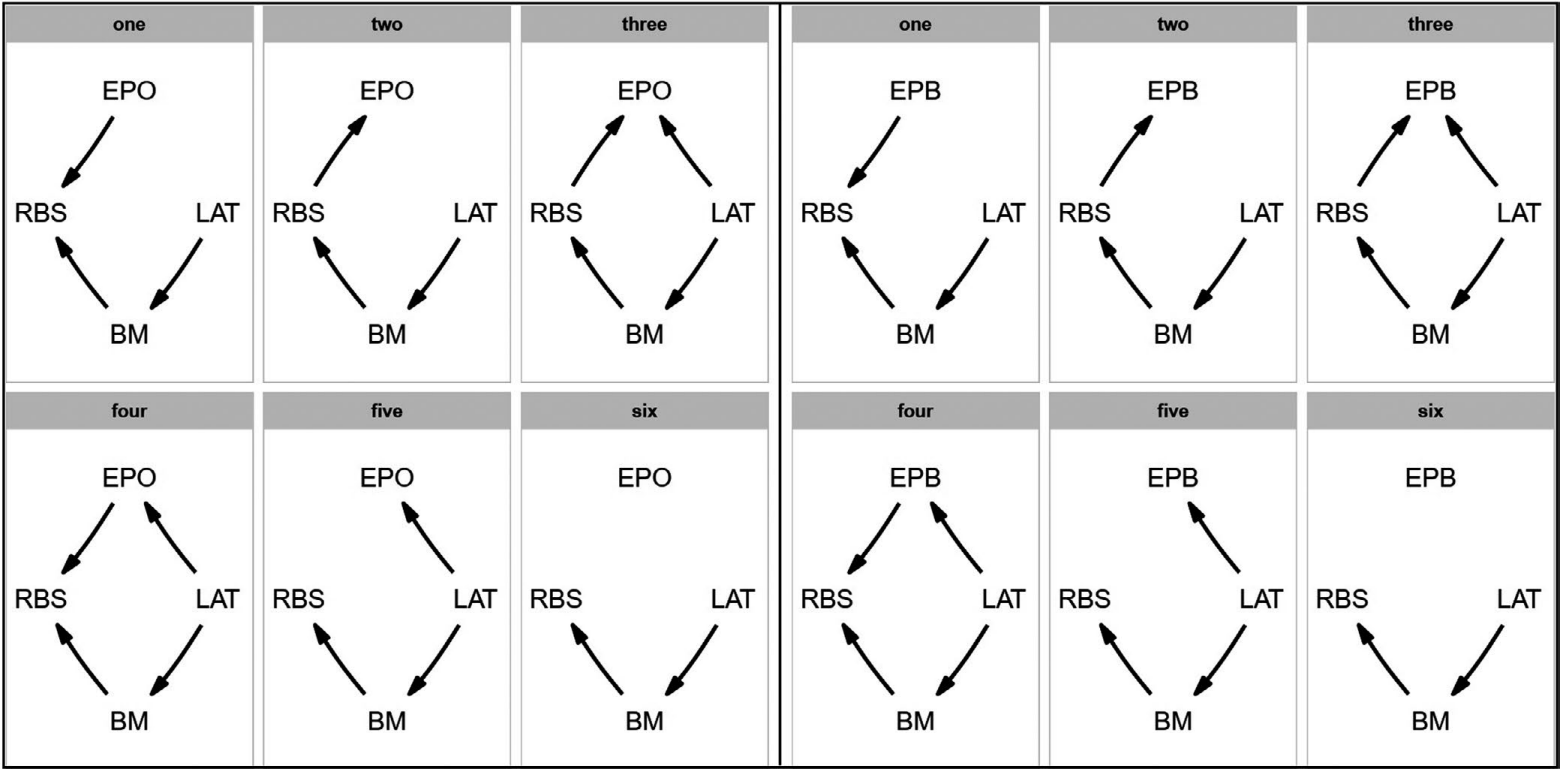
Alternative path models depicting the relationship between EPO rates (left six prespecified models for EPO, right six prespecified models for EPB), relative brain size (RBS), latitude (LAT), and body mass (BM)

Phylogenetic path analysis approach was used to deconstruct causal effects in the relationship between EPP rate and relative brain size (i.e., relative brain size affecting EPP rate, or the reverse or no causal link; Figure 1). We defined six possible causal models including body mass and latitude that could influence the relationship between EPP rate and relative brain size (Table S1). The fit of each model was tested using the d‐separation method (von Hardenberg & Voyer, 2013). The C‐statistic information criterion (CICc), corrected for small sample size, was used to discuss the importance of variables and directionality of effects. Models with ΔCICc values <2 are considered to have substantial support (Burnham & Anderson, 2002). The average model of the best‐performing models (ΔCICc < 2) was calculated when more than one substantial supported models existed (von Hardenberg & Voyer, 2013).

All statistical analyses were performed with R software (ver. 4.0). PGLS models were constructed using the caper package (Orme et al., 2013). Phylogenetic path analyses were conducted using the R package *phylopath* (von Hardenberg and Gonzalez‐Voyer 2013). We applied all these analyses through the summary tree. Precocial birds are assigned if they are able to move on their own soon after hatching and the rest as altricial birds (Sayol et al., 2018). For each test, we reported the mean estimates and two‐tailed significance values for each explanatory variable. Values were presented with mean ± standard error (*SE*) and two‐tailed significance set as 0.05 throughout the paper.

## 3 RESULTS

**FIGURE 2:**
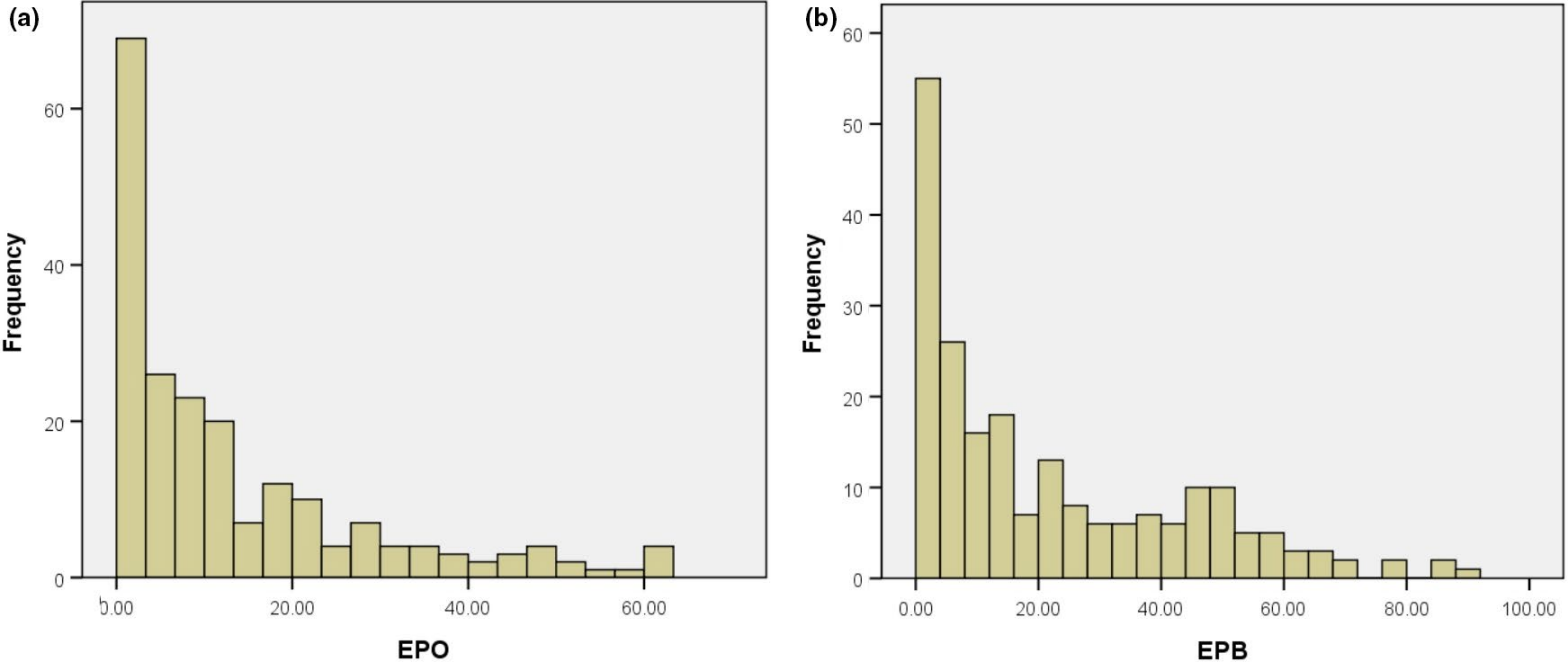
Distributions of (a) percentage extra‐pair offspring (EPO), and (b) percentage of broods with at least one extra‐pair offspring (EPB) for 215 species

We included data on EPP rate and relative brain size for 215 avian species from 73 families of 22 orders (63 precocial species and 152 altricial species; Table S1). We focused on socially monogamous pairs in species in our dataset and found that EPP was present in 80.4% (173/215) of these species. In 27.0% (58/215) of these species, the EPP was rare with less than 5% of broods contained EP offspring (Figure 1). Among these species, EPO averaged 12.97 ± 15.14% (*n* = 206 species) and EPB 21.71 ± 21.79% (*n* = 211 species) across species (Figure 2). The EPP level of precocial birds is much lower than that of altricial birds (EPO: 6.7 ± 8.8% versus. 15.4 ± 16.4%, *t*_204_ = 3.84, *p* < .001; EPB: 15.4 ± 20.5% versus. 24.3 ± 21.9%, *t*_209_ = 2.75, *p* = .006).

There were strong phylogenetic signals for EPP rate (EPO and EPB) in relation to relative brain size with latitude as a covariate; the *λ* values were significantly different from 0 (Table 1). Phylogenetically corrected analyses (PGLS) to examine the relationship between brain size and EPP rate when controlling the effect of latitude revealed that EPO was significantly linked with relative brain size, while the relationship between EPO and latitude is not statistically significant (Table 1). PGLS models yielded qualitatively equivalent results when EPB was a response variable (Table 1). Across 215 avian species, relative brain size was significantly and negatively correlated with EPP rate (both EPO and EPB), while the effect of latitude is not significant.

**TABLE 1:** Results of PGLS models to detect whether EPP rate was predicted by relative brain size and latitude

Model	Estimate ± *SE*	*t*	*p*	*λ* ^a^
Response variable: EPO (*n* = 206 species)				
Intercept	10.571 ± 6.568	1.610	.109	0.583^<.001, <.001^
Relative brain size	−13.733 ± 4.910	−2.797	.**006**	
Latitude	−0.124 ± 0.088	−1.417	.159	
Response variable: EPB (*n* = 211 species)				
Intercept	21.059 ± 8.856	2.378	.019	0.554^<.001, <.001^
Relative brain size	−19.856 ± 6.637	−2.992	.**003**	
Latitude	−0.159 ± 0.123	−1.298	.196	

Note

Analyses were run with 215 species; significant effects are shown in bold.

^a^ Superscripts following *λ* mean *p*‐values against models with *λ* = 0 and *λ* = 1, respectively.

**FIGURE 3:**
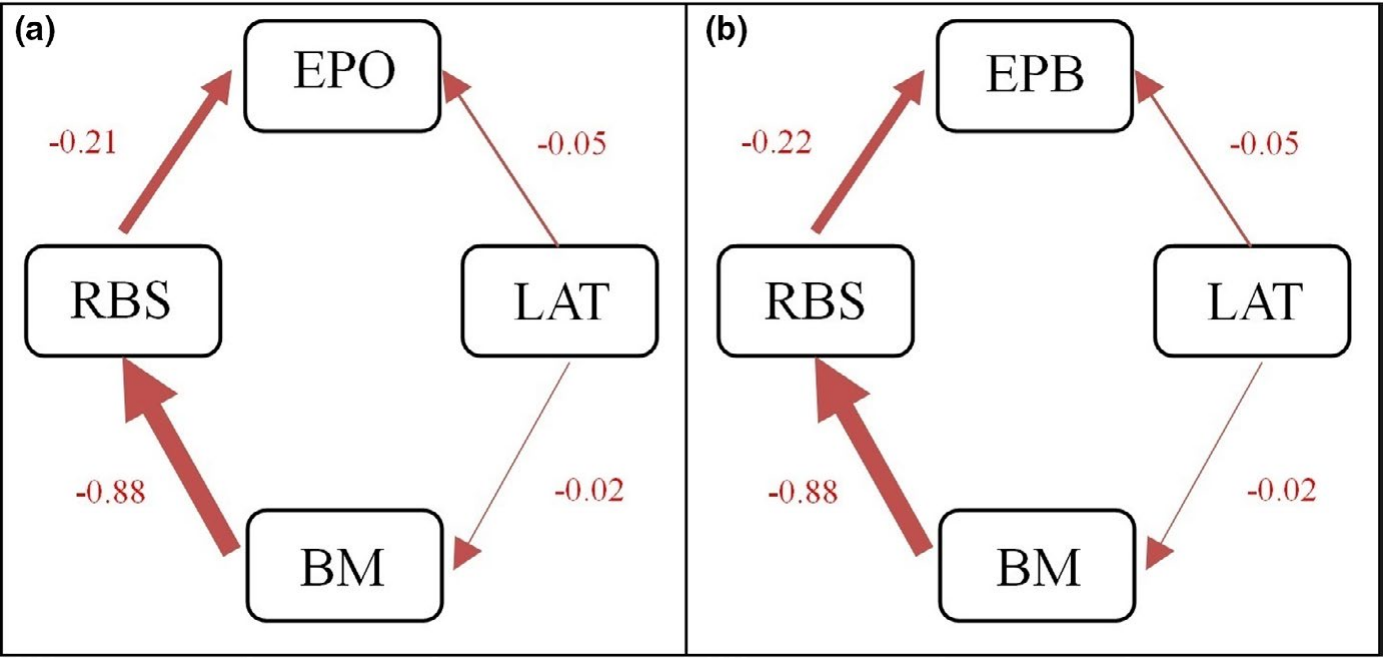
Averaged best‐fitting path models with ΔCICc ≤ 2 across 215 bird species. Arrows reflect the direction of the path, and their line width is proportional to their standardized regression coefficients (adjacent to arrows). The numbers on the arrows are regression coefficients, and the red lines indicate negative correlation (EPO and EPB included in models separately; RBS, relative brain size; LAT, latitude; BM, body mass)

**FIGURE 4:**
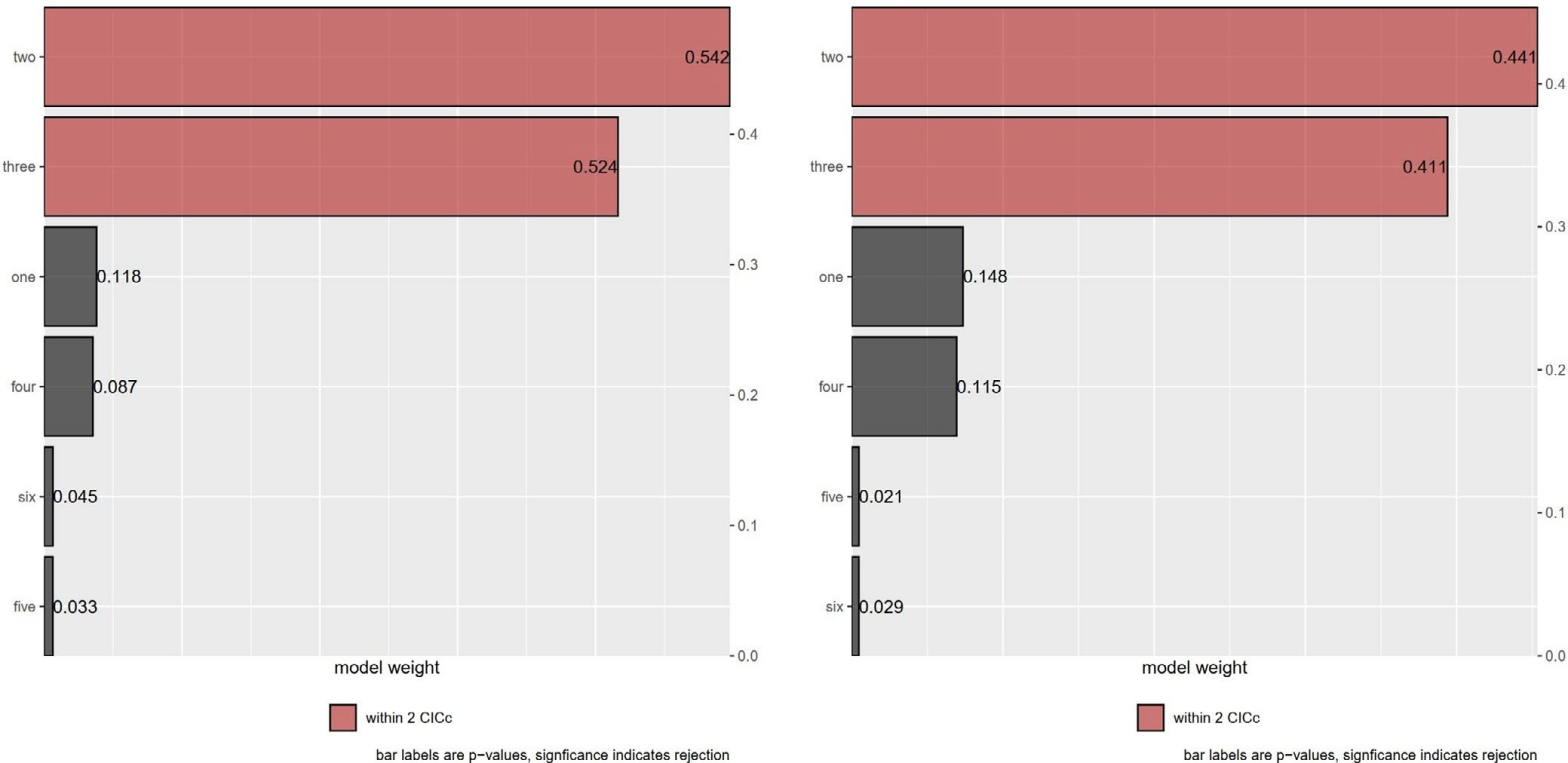
Relative importance of the six candidate causal models for EPO and EPB, respectively (left six models for EPO, right six models for EPB)

**FIGURE 5:**
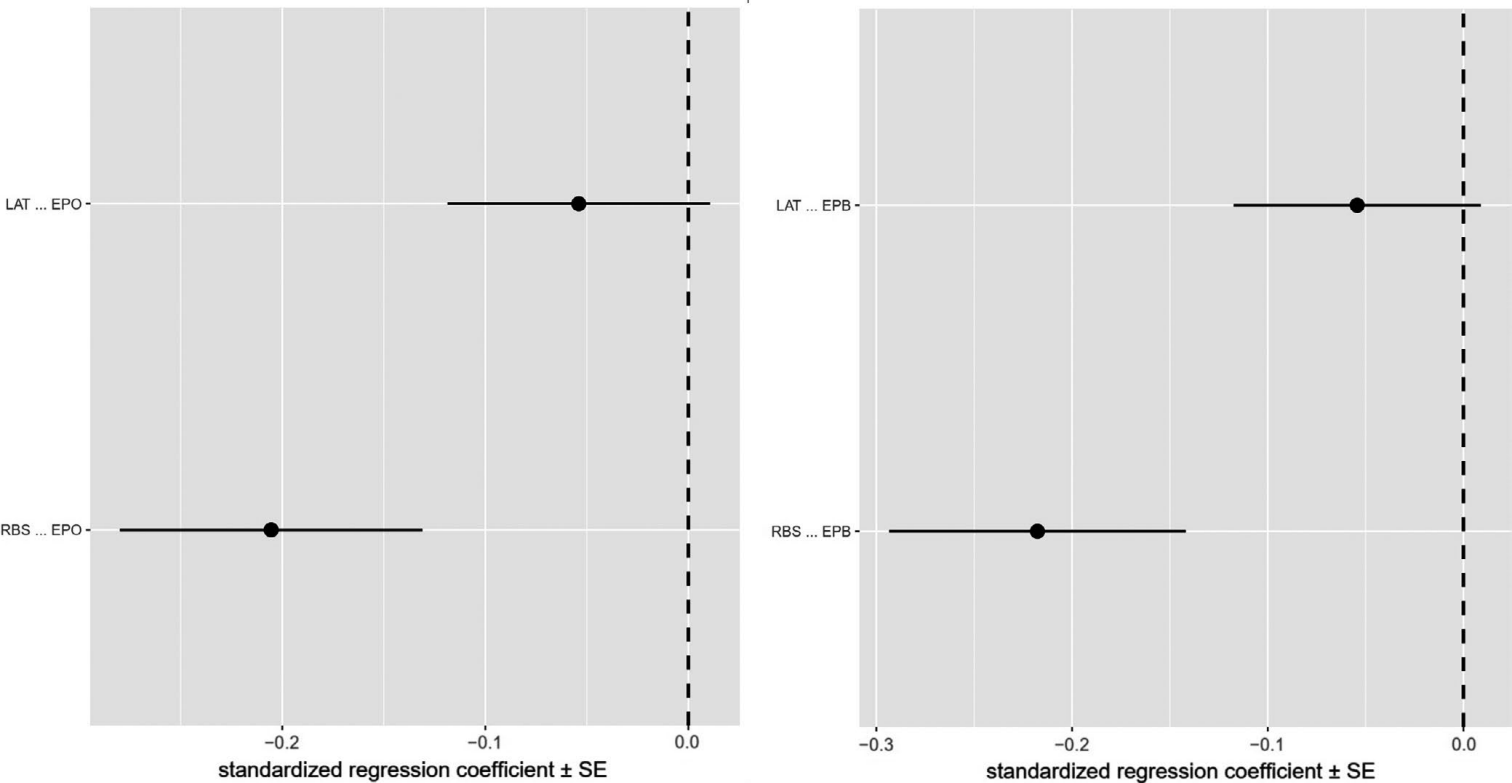
Standardized path coefficients and their standard errors for the averaged best‐fitting path model (left: EPO as the response; right: EPB as the response)

We analyzed alternative scenarios of potential causal relationships between EPP rate and relative brain size using phylogenetic path analysis (Figure 3). Similar results were reached from the six prespecified path models for EPO and EPB, respectively. Models II and III are the two best‐performing models (substantially supported models) with ΔCICc < 2 (Table 2, Figures 4 and 5). The averaged substantially supported models are also qualitatively equivalent when EPO or EPB was included (Figure 3). Two average models all reveal that the EPP rate is restricted by large brains (EPO ~ RBS: path coefficient = −0.21, *p* < .05; EPB ~ RBS: path coefficient = −0.22, *p* < .05; Figure 3).

**TABLE 2:** Results of association between EPO rates, relative brain size, latitude, and body mass using the phylogenetic path analyses, ranking the candidate models based on their CICc (the models with ΔCICc < 2 are represented in bold and were used to calculate the average model)

Model	*k*	*q*	*C*	*p*	CIC*c*	ΔCIC*c*	*W_i_*
EPO in PPA model							
**II**	**3**	**7**	**5.02**	.**542**	**19.6**	**0**	**0.497**
**III**	**2**	**8**	**3.21**	.**524**	**19.9**	**0.356**	**0.416**
I	3	7	10.15	.118	24.7	5.138	0.038
IV	2	8	8.13	.087	24.9	5.284	0.035
VI	4	6	15.85	.045	28.3	8.695	0.006
V	3	7	13.73	.033	28.3	8.717	0.006
EPB in PPA model							
**II**	**3**	**7**	**5.84**	.**441**	**20.4**	**0**	**0.454**
**III**	**2**	**8**	**3.96**	.**411**	**20.7**	**0.281**	**0.394**
I	3	7	9.48	.148	24	3.638	0.074
IV	2	8	7.44	.115	24.1	3.754	0.069
V	3	7	14.96	.021	29.5	9.114	0.005
VI	4	6	17.12	.029	29.5	9.135	0.005

## 4 DISCUSSION

In this study, we examined the effect of relative brain size on EPP patterns across 215 avian species using phylogenetic comparative analyses. We found that relative brain size was significantly and negatively linked to the EPP rate (both EPO and EPB). The most plausible causal scenario identified by our phylogenetic path analyses suggests that the EPP rate should be restricted by large brains. This result contrasted with our prediction that larger brains would be expected as EPP rate increased across avian species. The effect of latitude on EPP was not statistically significant, which is inconsistent with the results from some previous studies (Bonier et al., 2014; Brouwer et al., 2017), but is in line with a comparative analysis with larger samples of species (Brouwer & Griffith, 2019).

Procuring EPCs is a complicated and intelligent behavior in both sexes, which can contribute to high level of EPP within population. Such complicated and intelligent behavior is considered to be associated with large brains. However, our finding revealed that relative brain size negatively affects the EPP rate. One possible explanation is that pursuing EPC is a natural instinct in birds and large‐brained birds who are usually highly intelligent can constrain their mate from engaging in EPCs. Therefore, low EPP rates are reached in large‐brained birds. For example, large‐brained males may reduce the level of cuckoldry through mate guarding, territorial behavior, etc. (Garamszegi et al., 2005). Mate guarding and territory defense have been proposed as drivers of increased brain size between the sexes, and these behaviors can lead to decreased EPP for both sexes (Garamszegi et al., 2005). Species with large brains often show increased cognitive capacities (Benson‐Amram et al., 2016; Sol et al., 2016). Thus, in larger‐brained birds, unfaithful females are more likely to be punished by their mates with reducing care for the current brood, and thereafter, reduced EPP can be expected (Cockburn, 2004; Valera, 2003). Besides, species with more parental care tend to have larger brains (West, 2014). More parental care (especially male parental care) means less time for both sexes to seek EPCs, which can lead to a reduced EPP rate in the population. All these mechanisms are expected to lead to lower EPP rates in large‐brained birds. Therefore, species with larger brains tend to be more restrained by each other.

In this study, however, only 215 species with EPP data were included in the analyses, accounting for ca 2% of the total avian species of the world. Although the number of studies per year reporting EPP rates has remained steady since the 1990s, sample sizes are still relatively small and some clades have not been studied on EPP rate. Besides, most studies reporting EPP rates have been conducted in Europe or North America, while studies on species from either Africa or North Asia are rare (Brouwer & Griffith, 2019). Therefore, more species in more clades and geographic areas need to be added in further comparative analyses. Future investigations should also explore the relationship between EPP and brain size at an intraspecific level or at an interspecific level by comparative analyses with larger samples.

## CONFLICT OF INTEREST

The authors have no conflict of interest to declare.

## AUTHOR CONTRIBUTIONS

**Min Chen:** Conceptualization (equal); data curation (equal); formal analysis (equal). **Guopan Li:** Data curation (equal); formal analysis (equal); writing‐original draft (equal). **Jinlong Liu:** Data curation (equal); writing‐original draft (equal). **Shaobin Li:** Conceptualization (lead); data curation (equal); formal analysis (equal); writing‐original draft (lead).

## Supporting information

Table S1

## Data Availability

Supplementary materials (Table S1) are available from the Dryad Digital Repository: https://doi.org/10.5061/dryad.3bk3j9kkd. The R code used to perform analyses is available upon request.
