# Supplementary material for: Large brain size is associated with low extra‐pair paternity across bird species
Source: Ecol Evol. 2021 Sep 14;11(19):13601–8. doi: 10.1002/ece3.8087 (PMC8495782; doi:10.1002/ece3.8087)
Supplement: Supplementary file 1 — Table S1 [file ECE3-11-13601-s001.pdf]

Electronic Supplementary Material

# Large brain size is associated with low extra-pair paternity across bird species

Min Chen[1], Jinlong Liu[1], Guopan Li[1], Shaobin Li[1,2*]

[1]*College of Life Science, Yangtze University, Jingzhou, China, postcode 434025*

[2] *MOE Key Laboratory of Biodiversity and Ecology Engineering, Beijing Normal University, China, postcode 100875*

*Corresponding author. E-mail: shaobinlee@yahoo.com

For Review Only

Table S1. Species, EPO (percentage EP offspring), EPB (percentage broods with EP offspring), latitude (Abs.), brains size (mm$^3$), body mass (g) among 215 bird species from the references of Dunning (2008), Sayol et al. (2018), Brouwer & Griffith (2019), Jiménez-Ortega et al. (2020) and et al.

| Scientific name | Brain size | Body mass | EPO | EPB | Latitude | Relative brain size | Ref. |
|---|---|---|---|---|---|---|---|
| *Acanthiza_pusilla* | 0.45 | 8.15 | 6.2 | 11.9 | 35.27 | 0.054 | 1,2,3,4 |
| *Accipiter_cooperii* | 4.7 | 439 | 19.3 | 34.1 | 44.97 | -0.041 | 1,2,3,4 |
| *Acrocephalus_arundinaceus* | 0.91 | 26.75 | 6.6 | 7.733 | 56.00 | 0.030 | 1,2,3,4 |
| *Acrocephalus_schoenobaenus* | 0.45 | 10.8 | 8.1 | 22.93 | 54.02 | -0.118 | 1,2,3,4 |
| *Actitis_hypoleucos* | 0.86 | 48 | 11.6 | 19.25 | 56.27 | -0.384 | 1,2,3,4 |
| *Actitis_macularius* | 0.68 | 48.24 | 2.9 | 11.1 | 47.17 | -0.622 | 1,2,3,4 |
| *Aegithalos_concinnus* | 0.37 | 6.1 | 5.7 | 17.2 | 31.95 | 0.036 | 1,2,3,4 |
| *Aegolius_funereus* | 4.02 | 91.17 | 0 | 0 | 41.00 | 0.765 | 1,2,3,4 |
| *Agelaius_phoeniceus* | 1.69 | 65.49 | 29.24 | 49.65 | 40.23 | 0.101 | 1,2,3,4 |
| *Alauda_arvensis* | 0.96 | 37.48 | 20.5 | 26.9 | 51.17 | -0.123 | 1,2,3,4 |
| *Alectura_lathami* | 5.68 | 2340 | 27.7 | 63.6 | 27.85 | -0.877 | 1,2,3,4 |
| *Alle_alle* | 2.1 | 171 | 1.6 | 1.6 | 77.00 | -0.270 | 1,2,3,4 |
| *Ammodramus_savannarum* | 0.65 | 17.45 | 62.1 | 36.8 | 38.9 | -0.044 | 1,2,9 |
| *Anas_platyrhynchos* | 5.44 | 1110.47 | 9.3 | 48 | 49.15 | -0.464 | 1,2,3,4 |
| *Anas_strepera* | 3.9 | 889.52 | 4.2 | 27.6 | 48.62 | -0.661 | 1,2,3,4 |
| *Aphelocoma_coerulescens* | 2.85 | 77.15 | 0 | 0 | 27.17 | 0.523 | 1,2,3,4 |
| *Aphelocoma_ultramarina* | 3.61 | 131 | 27.85 | 50.7 | 31.90 | 0.435 | 1,2,3,4 |
| *Apus_apus* | 0.67 | 37.6 | 4.5 | 9.5 | 51.75 | -0.484 | 1,2,3,4 |
| *Asio_otus* | 5.31 | 209.68 | 0 | 0 | 46.88 | 0.533 | 1,2,3,4 |
| *Athene_cunicularia* | 3.78 | 150 | 1.7 | 1.5 | 38.72 | 0.398 | 1,2,3,4 |
| *Athene_noctua* | 3.7 | 169 | 0 | 0 | 51.27 | 0.304 | 1,2,3,4 |
| *Baeolophus_bicolor* | 1.01 | 21.6 | 8.8 | 22.2 | 40.00 | 0.266 | 1,2,3,4 |
| *Bartramia_longicauda* | 1.3 | 161.07 | 18.1 | 30 | 39.08 | -0.712 | 1,2,3,4 |
| *Branta_bernicla* | 6.1 | 1279.25 | 5.6 | NA | 61.25 | -0.436 | 1,2,3,4 |
| *Branta_canadensis* | 11.53 | 3178.8 | NA | 14.3 | 43.00 | -0.357 | 1,2,3,4 |

| | | | | | | | |
|---|---|---|---|---|---|---|---|
| *Buteo_galapagoensis* | 8.3 | 1163.33 | 0 | 0 | 0.23 | -0.070 | 1,2,3,4 |
| *Buteo_swainsoni* | 7.36 | 1064 | 5.4 | 7.4 | 41.82 | -0.135 | 1,2,3,4 |
| *Calamospiza_melanocorys* | 1.18 | 37.6 | 25.1 | 52.3 | 41.35 | 0.082 | 1,2,3 |
| *Calidris_alba* | 1.01 | 57 | 2 | 6.3 | 74.50 | -0.329 | 1,2,3,4 |
| *Calidris_mauri* | 0.64 | 27.85 | 6.6 | 8 | 64.33 | -0.346 | 1,2,3,4 |
| *Calonectris_diomedea* | 4.92 | 571.83 | 3.867 | 6.067 | 37.97 | -0.158 | 1,2,3 |
| *Campylorhynchus_griseus* | 1.31 | 42.4 | 2.3 | NA | 8.50 | 0.113 | 1,2,3,4 |
| *Cardinalis_cardinalis* | 1.52 | 42.65 | 13.5 | 15.8 | 37.65 | 0.258 | 1,2,3 |
| *Carduelis_tristis* | 0.54 | 12.8 | 14.3 | 26.7 | 43.53 | -0.040 | 1,2,3,4 |
| *Carpodacus_mexicanus* | 0.78 | 21.4 | 7.333 | 13.97 | 45.32 | 0.013 | 1,2,3,4 |
| *Catharacta_maccormicki* | 6.79 | 1349 | 7.1 | 7.7 | 77.52 | -0.361 | 1,2,3,4 |
| *Centrocercus_urophasianus* | 4 | 2208.75 | NA | 13.95 | 43.53 | -1.192 | 1,2,3,4 |
| *Centropus_phasianinus* | 4.12 | 389.5 | 18.6 | 47.6 | 12.43 | -0.100 | 1,2,3,4 |
| *Cepphus_grylle* | 3.61 | 378 | 0 | 0 | 67.43 | -0.214 | 1,2,3,4 |
| *Cercomacra_tyrannina* | 0.74 | 16.3 | 0 | 0 | 9.12 | 0.127 | 1,2,3 |
| *Charadrius_alexandrinus* | 0.89 | 42.3 | 0.883 | 1.717 | 27.90 | -0.272 | 1,2,3,4 |
| *Charadrius_falklandicus* | 1.11 | 65 | 0 | 0 | 52.43 | -0.315 | 1,2,3,4 |
| *Charadrius_modestus* | 1.24 | 78 | 0 | 0 | 52.43 | -0.316 | 1,2,3,4 |
| *Charadrius_pecuarius* | 0.82 | 34 | 0 | 0 | 22.03 | -0.221 | 1,2,3,4 |
| *Charadrius_ruficapillus* | 0.82 | 37.4 | 0 | 0 | 37.88 | -0.279 | 1,2,3,4 |
| *Charadrius_semipalmatus* | 0.96 | 46.71 | 4.7 | 4.2 | 58.75 | -0.257 | 1,2,3,4 |
| *Chen_caerulescens* | 10.68 | 2641 | 5 | 13 | 67.23 | -0.320 | 1,2,3,4 |
| *Chen_rossii* | 6.85 | 1636 | 2.4 | 8.3 | 67.23 | -0.470 | 1,2,3,4 |
| *Chlidonias_hybrida* | 1.48 | 83.9 | 8.1 | 11.8 | 51.73 | -0.183 | 1,2,3,4 |
| *Chrysococcyx_basalis* | 0.62 | 23.15 | 0 | 0 | 35.27 | -0.265 | 1,2,3,4 |
| *Chthonicola_sagittatus* | 0.6 | 13.5 | 10.7 | 12.5 | 35.27 | 0.033 | 1,2,3,4 |
| *Ciconia_ciconia* | 14.67 | 3448 | NA | 26.9 | 44.00 | -0.166 | 1,2,3,4 |
| *Clamator_glandarius* | 1.9 | 143.75 | NA | 59.1 | 37.18 | -0.263 | 1,2,3,4 |
| *Colaptes_auratus* | 3.06 | 126.5 | 1.2 | 7.15 | 52.37 | 0.292 | 1,2,3,4 |
| *Colinus_virginianus* | 1.3 | 166 | NA | 85.3 | 36.00 | -0.731 | 1,2,3,4 |
| *Coracias_garrulus* | 2.32 | 146 | 5.3 | 4.1 | 37.30 | -0.073 | 1,2,3,4 |
| *Coragyps_atratus* | 11.99 | 2080.5 | 0 | 0 | 35.75 | -0.058 | 1,2,3,4 |
| *Corcorax_melanorhamphos* | 5.2 | 349.1 | 0 | 0 | 35.27 | 0.200 | 1,2,3,4 |
| *Corvus_brachyrhynchos* | 7.17 | 438.54 | 10.4 | 21.7 | 42.40 | 0.382 | 1,2,3,4 |
| *Corvus_corone* | 8.51 | 485.9 | NA | 15.8 | 42.00 | 0.490 | 1,2,3,4 |
| *Corvus_monedula* | 5.15 | 246 | 1.3 | 3.35 | 51.78 | 0.405 | 1,2,3,4 |
| *Cuculus_canorus* | 1.57 | 111.5 | NA | 14.3 | 36.00 | -0.298 | 1,2,3,4 |
| *Cyanocitta_stelleri* | 3.54 | 128 | 15.2 | 14.6 | 40.98 | 0.430 | 1,2,3,4 |
| *Cyanocorax_morio* | 4.8 | 204 | 16.81 | 21.62 | 10.20 | 0.449 | 1,2,3,4 |
| *Cyanoliseus_patagonus* | 8.23 | 278 | 0 | 0 | 41.05 | 0.799 | 1,2,3,4 |
| *Cygnus_atratus* | 12.15 | 5650 | 15.1 | 37.6 | 37.55 | -0.656 | 1,2,3,4 |
| *Dacelo_novaeguineae* | 4.31 | 334.5 | 0 | 0 | 35.27 | 0.038 | 1,2,3,4 |
| *Delichon_urbicum* | 0.48 | 14.5 | 18.85 | 33.3 | 58.76 | -0.234 | 1,2,3,4 |
| *Dendroica_caerulescens* | 0.43 | 10.15 | 30.6 | 44.03 | 43.93 | -0.126 | 1,2,3,4 |

| | | | | | | | |
|---|---|---|---|---|---|---|---|
| *Dendroica_petechia* | 0.52 | 11.07 | 28.4 | 46.7 | 46.35 | 0.011 | 1,2,3,4 |
| *Diomedea_exulans* | 28.25 | 7046.67 | 13 | 12.35 | 49.13 | 0.052 | 1,2,3 |
| *Dromaius_novaehollandiae* | 28.88 | 34200 | 50.9 | 88.9 | 32.22 | -0.893 | 1,2,3,4 |
| *Dumetella_carolinensis* | 1.14 | 37.8 | 13 | 24.8 | 39.00 | 0.044 | 1,2,3,4 |
| *Eclectus_roratus* | 7.36 | 561 | 8.1 | 8.1 | 12.75 | 0.257 | 1,2,3,4 |
| *Empidonax_minimus* | 0.37 | 10.48 | 34.9 | 56.5 | 44.57 | -0.296 | 1,2,3,4 |
| *Empidonax_virescens* | 0.43 | 12.9 | 37.17 | 49.27 | 42.08 | -0.273 | 1,2,3,4 |
| *Erythropygia_coryphaeus* | 0.72 | 23.05 | 18.2 | 33.3 | 33.68 | -0.113 | 1,2,3 |
| *Erythrura_gouldiae* | 0.53 | 10 | 8.6 | 22.8 | 15.57 | 0.093 | 1,2,3,4 |
| *Eudyptes_schlegeli* | 18.5 | 4250 | 3.8 | 7.7 | 54.50 | -0.062 | 1,2,3,4 |
| *Euplectes_orix* | 0.76 | 23.1 | 28.35 | 47.15 | 33.43 | -0.060 | 1,2,3,4 |
| *Falco_columbarius* | 3.09 | 157.39 | 0 | 0 | 52.12 | 0.167 | 1,2,3,4 |
| *Falco_eleonorae* | 4.04 | 385 | 0 | 0 | 38.00 | -0.112 | 1,2,3,4 |
| *Falco_naumanni* | 2.71 | 152.5 | 5.3 | 8.333 | 39.96 | 0.056 | 1,2,3,4 |
| *Falco_peregrinus* | 6.19 | 743.07 | 0 | 0 | 63.00 | -0.088 | 1,2,3,4 |
| *Falco_sparverius* | 2.49 | 91.98 | 11.2 | 9.5 | 42.42 | 0.281 | 1,2,3,4 |
| *Falco_tinnunculus* | 3.87 | 184 | 1.9 | 2.7 | 62.98 | 0.297 | 1,2,3,4 |
| *Ficedula_albicollis* | 0.45 | 12.7 | 20.53 | 40.93 | 50.81 | -0.217 | 1,2,3 |
| *Forpus_passerinus* | 1.1 | 23 | 7.7 | 14.4 | 8.57 | 0.313 | 1,2,3,4 |
| *Fratercula_arctica* | 4.05 | 483.5 | 0 | 0 | 67.43 | -0.249 | 1,2,3,4 |
| *Fregata_minor* | 9.23 | 1296 | 4.9 | 4.9 | 23.75 | -0.030 | 1,2,3 |
| *Fringilla_coelebs* | 0.81 | 23.78 | 17 | 23.1 | 53.33 | -0.014 | 1,2,3,4 |
| *Fulmarus_glacialis* | 6.33 | 613 | 0 | 0 | 59.53 | 0.052 | 1,2,3 |
| *Furnarius_rufus* | 1.33 | 39.9 | 3.33 | 6.52 | 15.76 | 0.165 | 1,2,10 |
| *Gallinula_chloropus* | 2.4 | 351 | 0 | 0 | 52.63 | -0.576 | 1,2,3,4 |
| *Gavia_immer* | 10.1 | 3093.78 | 0 | 0 | 45.70 | -0.472 | 1,2,3,4 |
| *Geothlypis_trichas* | 0.52 | 9.43 | 20.07 | 45.57 | 43.38 | 0.109 | 1,2,3,4 |
| *Gerygone_flavolateralis* | 0.34 | 6.4 | 8.7 | 16.7 | 30.8 | -0.078 | 1,2,8 |
| *Grallina_cyanoleuca* | 1.68 | 82.78 | 2.9 | 6.4 | 35.27 | -0.048 | 1,2,3,4 |
| *Grus_canadensis* | 14.82 | 3901.04 | 11.1 | 22.2 | 43.60 | -0.231 | 1,2,3 |
| *Guira_guira* | 1.92 | 141 | 11.1 | NA | 15.78 | -0.241 | 1,2,3,4 |
| *Gymnorhina_tibicen* | 4.65 | 286.5 | 62.85 | 86.4 | 34.81 | 0.209 | 1,2,3,4 |
| *Habia_fuscicauda* | 1.34 | 39.7 | 41.5 | 52.6 | 9.08 | 0.176 | 1,2,3 |
| *Haematopus_ostralegus* | 4.52 | 514.5 | 1.5 | 3.8 | 53.48 | -0.178 | 1,2,3,4 |
| *Haliaeetus_albicilla* | 16.5 | 4793 | 0 | 0 | 49.3 | -0.250 | 1,2,5 |
| *Hirundo_ariel* | 0.36 | 10.85 | 13.8 | 20 | 37.80 | -0.344 | 1,2,3,4 |
| *Hirundo_rustica* | 0.55 | 19.3 | 23.63 | 43.24 | 43.53 | -0.273 | 1,2,3,4 |
| *Hylocichla_mustelina* | 1.25 | 50.15 | 23.1 | 40.2 | 42.51 | -0.037 | 1,2,3,4 |
| *Icteria_virens* | 0.91 | 24.9 | 30.8 | 50.9 | 43.45 | 0.074 | 1,2,3,4 |
| *Icterus_galbula* | 1.13 | 33.27 | 32.2 | 45.8 | 36.37 | 0.113 | 1,2,3,4 |
| *Irediparra_gallinacea* | 1.13 | 102.55 | 2.9 | 10 | 19.57 | -0.576 | 1,2,3,4 |
| *Jabiru_mycteria* | 23.5 | 6054.5 | 2.9 | 7.7 | 17.00 | -0.039 | 1,2,3,4 |
| *Junco_hyemalis* | 0.83 | 20.1 | 29.52 | 39.05 | 35.87 | 0.113 | 1,2,3 |
| *Jynx_torquilla* | 0.94 | 36.5 | 0.7 | 2 | 51.90 | -0.127 | 1,2,3,4 |

For Review Only

| | | | | | | | |
|---|---|---|---|---|---|---|---|
| *Lagopus_lagopus* | 2.38 | 459.56 | 9.4 | 13.2 | 59.83 | -0.750 | 1,2,3,4 |
| *Lanius_collurio* | 0.96 | 28.45 | 5.3 | 16.7 | 46.20 | 0.046 | 1,2,3,4 |
| *Lanius_ludovicianus* | 1.53 | 51.75 | 4.5 | 13.9 | 34.00 | 0.146 | 1,2,3,4 |
| *Larus_canus* | 4.07 | 415.75 | 3.6 | 8.3 | 51.67 | -0.152 | 1,2,3,4 |
| *Larus_occidentalis* | 6.89 | 931.5 | 0 | 0 | 37.70 | -0.120 | 1,2,3,4 |
| *Larus_ridibundus* | 3.05 | 276.9 | 20.3 | 33.3 | 48.92 | -0.192 | 1,2,3,4 |
| *Lichenostomus_chrysops* | 0.72 | 17.5 | 44.4 | NA | 37.68 | 0.056 | 1,2,3,4 |
| *Locustella_luscinioides* | 0.55 | 15 | 4.1 | 5.9 | 40.63 | -0.119 | 1,2,3,4 |
| *Loxia_curvirostra* | 1.42 | 34.3 | 0 | 0 | 59.50 | 0.323 | 1,2,3,4 |
| *Luscinia_megarhynchos* | 0.7 | 19.4 | 21.5 | 46.4 | 52.40 | -0.035 | 1,2,3 |
| *Malurus_coronatus* | 0.475 | 9 | 5.7 | 6.9 | 17.52 | 0.047 | 1,2,3 |
| *Malurus_cyaneus* | 0.47 | 9.3 | 63.29 | 76.36 | 35.78 | 0.017 | 1,2,3 |
| *Malurus_elegans* | 0.57 | 10.08 | 60.4 | 70.3 | 34.35 | 0.160 | 1,2,3 |
| *Malurus_melanocephalus* | 0.45 | 7.9 | 50.55 | 64.35 | 22.32 | 0.073 | 1,2,3 |
| *Malurus_splendens* | 0.45 | 9.43 | 42.2 | 55.3 | 34.33 | -0.035 | 1,2,3 |
| *Manorina_melanocephala* | 1.86 | 60.3 | 5.9 | 5.7 | 27.33 | 0.248 | 1,2,3,4 |
| *Manorina_melanophrys* | 1.14 | 30.45 | 4.2 | 7.7 | 37.68 | 0.176 | 1,2,3,4 |
| *Megascops_asio* | 4.91 | 180.5 | 0 | 0 | 37.62 | 0.547 | 1,2,3,4 |
| *Melanerpes_formicivorus* | 1.99 | 79.65 | 0 | 0 | 36.37 | 0.145 | 1,2,3,4 |
| *Meleagris_gallopavo* | 8.21 | 6050 | 11.6 | 45.2 | 36.37 | -1.090 | 1,2,3,4 |
| *Melospiza_georgiana* | 0.81 | 17.6 | 20.85 | 41.5 | 39.45 | 0.170 | 1,2,3 |
| *Molothrus_ater* | 1.15 | 41.67 | 4.7 | 21.45 | 50.18 | -0.007 | 1,2,3,4 |
| *Monias_benschi* | 2 | 138 | 11.8 | 18.2 | 23.07 | -0.187 | 1,2,3,4 |
| *Mycteria_americana* | 14.88 | 2558.5 | 0 | 0 | 14.00 | 0.031 | 1,2,3,4 |
| *Myiopsitta_monachus* | 4.08 | 120 | 0 | 11.38 | 32.79 | 0.612 | 1,2,3,4 |
| *Oceanites_oceanicus* | 0.71 | 33.7 | 0 | 0 | 51.72 | -0.359 | 1,2,3 |
| *Oceanodroma_leucorhoa* | 0.89 | 36.93 | 0 | 0 | 44.58 | -0.189 | 1,2,3 |
| *Oenanthe_oenanthe* | 0.78 | 30.6 | 13.33 | 28.6 | 51.68 | -0.206 | 1,2,3 |
| *Pachycephala_pectoralis* | 1.12 | 25.4 | 19.2 | 23.1 | 37.52 | 0.270 | 1,2,3,4 |
| *Pachyptila_belcheri* | 2 | 92.6 | 20.6 | 20.6 | 51.72 | 0.057 | 1,2,3 |
| *Parus_atricapillus* | 0.76 | 12 | 11.85 | 31.3 | 44.57 | 0.341 | 1,2,3,4 |
| *Parus_gambeli* | 0.75 | 11.4 | 17.9 | 43.2 | 50.7 | 0.359 | 1,2,6 |
| *Passer_domesticus* | 0.97 | 24.3 | 13.46 | 30.83 | 44.01 | 0.153 | 1,2,3,4 |
| *Passerculus_sandwichensis* | 0.68 | 26 | 43.76 | 63.34 | 44.35 | -0.243 | 1,2,3 |
| *Passerina_cyanea* | 0.65 | 14.7 | 34.9 | 48 | 35.88 | 0.061 | 1,2,3 |
| *Petroica_goodenovii* | 0.37 | 8.9 | 22.1 | 37 | 36.17 | -0.195 | 1,2,3 |
| *Phainopepla_nitens* | 0.79 | 22.1 | 0 | 0 | 33.68 | 0.006 | 1,2,3,4 |
| *Phalacrocorax_atriceps* | 14.13 | 3050 | 0 | 0 | 43.08 | -0.128 | 1,2,3 |
| *Phalacrocorax_carbo* | 10.45 | 2571.17 | 10.5 | 30 | 51.73 | -0.325 | 1,2,3 |
| *Phalaropus_fulicarius* | 0.56 | 49.4 | 8.6 | 33.3 | 69.40 | -0.831 | 1,2,3,4 |
| *Phalaropus_lobatus* | 0.45 | 35.68 | 1.8 | 6.3 | 66.56 | -0.850 | 1,2,3,4 |
| *Philomachus_pugnax* | 1.56 | 136 | NA | 50.75 | 59.00 | -0.427 | 1,2,3,4 |
| *Phoenicurus_phoenicurus* | 0.51 | 14.6 | 2 | 10.5 | 62.47 | -0.178 | 1,2,3 |
| *Phylloscopus_sibilatrix* | 0.36 | 7.16 | 8.35 | 16.65 | 47.42 | -0.090 | 1,2,3,4 |

For Review Only

| | | | | | | | |
|---|---|---|---|---|---|---|---|
| *Picoides_borealis* | 1.66 | 48 | 1.3 | 2.3 | 35.52 | 0.273 | 1,2,3,4 |
| *Picoides_tridactylus* | 2.63 | 65.65 | 4.9 | 11.55 | 54.36 | 0.542 | 1,2,3,4 |
| *Platycercus_elegans* | 3.78 | 124.5 | 0 | 0 | 33.7 | 0.513 | 1,2,11 |
| *Plectrophenax_nivalis* | 1.02 | 42.2 | 10.8 | 20.9 | 78.22 | -0.135 | 1,2,3,4 |
| *Plocepasser_mahali* | 1.23 | 43.3 | 11.6 | 13.3 | 27.27 | 0.037 | 1,2,3,4 |
| *Pluvialis_dominica* | 1.82 | 149.19 | 7.6 | 16.2 | 71.29 | -0.329 | 1,2,3,4 |
| *Poephila_acuticauda* | 0.51 | 14 | 12.8 | 25.7 | 15.55 | -0.152 | 1,2,3,4 |
| *Pomatostomus_temporalis* | 1.9 | 75 | 18.8 | 26.8 | 30.88 | 0.135 | 1,2,3 |
| *Porphyrio_hochstetteri* | 8.4 | 2470.5 | 0 | 0 | 38.00 | -0.519 | 1,2,3,4 |
| *Porphyrio_porphyrio* | 4.7 | 744.13 | 0 | 0 | 45.93 | -0.365 | 1,2,3,4 |
| *Progne_subis* | 1.03 | 53.8 | 20.45 | 46.1 | 40.61 | -0.274 | 1,2,3,4 |
| *Prunella_modularis* | 0.78 | 19.7 | 8.9 | 14.35 | 49.03 | 0.064 | 1,2,3 |
| *Psaltriparus_minimus* | 0.35 | 5.3 | 0 | 0 | 31.85 | 0.066 | 1,2,3,4 |
| *Puffinus_tenuirostris* | 4.5 | 556.7 | 10.8 | 10.8 | 43.10 | -0.230 | 1,2,3 |
| *Pygoscelis_adeliae* | 19.66 | 4850 | 9.1 | 11.1 | 74.35 | -0.082 | 1,2,3,4 |
| *Pyrocephalus_rubinus* | 0.46 | 14.4 | 47.1 | 63.6 | 19.28 | -0.272 | 1,2,3,4 |
| *Rhipidura_fuliginosa* | 0.34 | 7.1 | 55.1 | 64 | 35.45 | -0.142 | 1,2,3,4 |
| *Riparia_riparia* | 0.41 | 13.45 | 17 | 36.1 | 52.25 | -0.346 | 1,2,3,4 |
| *Rissa_tridactyla* | 4.31 | 387.25 | 0 | 0 | 48.08 | -0.051 | 1,2,3,4 |
| *Sayornis_phoebe* | 0.59 | 18.3 | 8.45 | 14.6 | 41.73 | -0.170 | 1,2,3,4 |
| *Sericornis_frontalis* | 0.76 | 11.4 | 12.4 | 23.5 | 35.27 | 0.373 | 1,2,3,4 |
| *Setophaga_ruticilla* | 0.36 | 8.25 | 35.05 | 54.1 | 43.99 | -0.176 | 1,2,3,4 |
| *Sialia_mexicana* | 0.84 | 26.45 | 25.37 | 51.18 | 36.25 | -0.043 | 1,2,3,4 |
| *Sialia_sialis* | 0.99 | 27.5 | 9.75 | 25.2 | 37.89 | 0.098 | 1,2,3,4 |
| *Sicalis_flaveola* | 0.7 | 15.4 | 31.8 | 51.8 | 35.6 | 0.106 | 1,2,7 |
| *Spheniscus_humboldti* | 15.98 | 4379 | 0 | 0 | 15.37 | -0.226 | 1,2,3,4 |
| *Spiza_americana* | 0.91 | 26.28 | 38.5 | 52.2 | 39.08 | 0.041 | 1,2,3 |
| *Steganopus_tricolor* | 0.65 | 59.95 | 0 | 0 | 51.45 | -0.800 | 1,2,3,4 |
| *Sterna_hirundo* | 1.88 | 139 | 0.5 | 1.45 | 49.43 | -0.253 | 1,2,3,4 |
| *Stipiturus_malachurus* | 0.4 | 7.3 | 12 | 14.8 | 38.38 | 0.004 | 1,2,3 |
| *Strix_aluco* | 9.08 | 475 | 0.7 | 2.7 | 46.00 | 0.569 | 1,2,3,4 |
| *Struthidea_cinerea* | 2.98 | 132 | 0 | 1.3 | 32.78 | 0.239 | 1,2,3,4 |
| *Sturnus_vulgaris* | 1.97 | 86 | 12.78 | 36.18 | 52.29 | 0.088 | 1,2,3,4 |
| *Tachycineta_bicolor* | 0.55 | 21.2 | 48.98 | 77.52 | 44.34 | -0.331 | 1,2,3,4 |
| *Taeniopygia_guttata* | 0.44 | 12.05 | 2 | 6.5 | 33.62 | -0.208 | 1,2,3,4 |
| *Tetrao_tetrix* | 3.8 | 1082.5 | 0 | 1.9 | 62.58 | -0.807 | 1,2,3,4 |
| *Thalassarche_cauta* | 19.34 | 3935 | 6.9 | 6.9 | 40.38 | 0.030 | 1,2,3 |
| *Thalassarche_chrysostoma* | 19.15 | 3507.5 | 7.2 | 6.7 | 54.00 | 0.090 | 1,2,3 |
| *Thalassarche_melanophrys* | 17.88 | 3232 | 5.7 | 5.6 | 54.00 | 0.072 | 1,2,3 |
| *Thalassoica_antarctica* | 6.68 | 688 | 7.3 | 7.3 | 71.88 | 0.035 | 1,2,3 |
| *Thryothorus_ludovicianus* | 0.85 | 17.2 | 0 | 0 | 34.82 | 0.233 | 1,2,3,4 |
| *Tinamus_major* | 2.89 | 1028.5 | 24 | 57.1 | 10.43 | -1.049 | 1,2,3,4 |
| *Troglodytes_aedon* | 0.53 | 10.4 | 13.7 | 37.02 | 42.04 | 0.069 | 1,2,3,4 |
| *Tryngites_subruficollis* | 1.03 | 62.4 | NA | 40.4 | 70.20 | -0.364 | 1,2,3,4 |

For Review Only

| | | | | | | | |
|---|---|---|---|---|---|---|---|
| *Turdus_migratorius* | 1.64 | 78.5 | 48.1 | 71.9 | 40.03 | -0.040 | 1,2,3,4 |
| *Tyrannus_forficatus* | 0.86 | 39.3 | 48.8 | 65.9 | 34.77 | -0.262 | 1,2,3,4 |
| *Tyrannus_tyrannus* | 0.95 | 39.93 | 43.37 | 59.5 | 43.10 | -0.172 | 1,2,3,4 |
| *Tyto_alba* | 6.51 | 355 | 0.9 | 2 | 46.82 | 0.414 | 1,2,3 |
| *Upupa_epops* | 1.35 | 66.82 | 5.5 | 15.5 | 41.67 | -0.136 | 1,2,3,4 |
| *Uria_aalge* | 5.73 | 991 | 7.8 | 7.8 | 51.75 | -0.342 | 1,2,3,4 |
| *Uria_lomvia* | 5.3 | 990.2 | 7.4 | 7.4 | 62.00 | -0.419 | 1,2,3,4 |
| *Vireo_griseus* | 0.58 | 11.4 | 2 | 5.6 | 31.40 | 0.102 | 1,2,3,4 |
| *Vireo_olivaceus* | 0.61 | 19.57 | 57.9 | 57.1 | 41.00 | -0.178 | 1,2,3,4 |
| *Volatinia_jacarina* | 0.49 | 9.7 | 35.6 | 47.05 | 15.94 | 0.033 | 1,2,3,4 |
| *Wilsonia_citrina* | 0.48 | 10.55 | 26.7 | 35.3 | 41.00 | -0.039 | 1,2,3,4 |
| *Zonotrichia_albicollis* | 1.05 | 24.4 | 16.7 | 28.95 | 44.15 | 0.230 | 1,2,3 |
| *Zonotrichia_leucophrys* | 0.97 | 28.12 | 39.13 | 49.27 | 42.08 | 0.064 | 1,2,3 |
| *Zosterops_lateralis* | 0.47 | 13.7 | 0 | 0 | 23.43 | -0.220 | 1,2,3,4 |

For Review Only

## References

[1]. Dunning, J. 2008. CRC Handbook of Avian Body Masses. CRC Press, Boca Raton, FL.

[2]. Brouwer, L. & Griffith, S. C. 2019. Extra-pair paternity in birds. Mol. Ecol. 28: 4864-4882.

[3]. Sayol, F., Downing, P.A., Iwaniuk, A.N., Maspons, J. & Sol, D. 2018. Predictable evolution towards larger brains in birds colonizing oceanic islands. Nat. Commun. 9: 2820.

[4]. Jiménez-Ortega D, Kolm N, Immler S, Maklakov AA, Gonzalez-Voyer A. Long life evolves in large brained bird lineages. Evolution. 2020, 74:2617-28.

[5]. Rymeová, D., Pavlíek, D., Kirner, J., Mráz, J. & Literák, I. 2020. Parentage analysis in the white-tailed eagle *Haliaeetus albicilla*: are moulted feathers from nest sites a reliable source of parental DNA? Acta Ornithol. 55(1): 41-52.

[6]. Bonderud, E. S., Otter, K. A., Burg, T. M., Marini, K. L. D., Reudink, M. W. & Hebets, E. 2018. Patterns of extra-pair paternity in mountain chickadees. Ethology 124: 378-386.

[7]. Saldívar, M. J. B., Mio, C. I. & Massoni, V. 2019. Genetic mating system, population genetics and effective size of Saffron Finches breeding in southern South America. Genetica 147:315–326.

[8]. Bojarska, K., Kuehn, R., Gazda, M. A., Sato, N. J., et al. 2018. Mating system and extra-pair paternity in the fan-tailed gerygone *Gerygone flavolateralis* in relation to parasitism by the shining bronze-cuckoo *chalcites lucidus*. PLoS ONE, 13(3): e0194059.

[9]. Danner, J. E., Small, D. M., Ryder, T. B., Lohr, B., Masters, B. S., Gill, D. E. & Fleischer, R. C. 2018. Temporal patterns of extra-pair paternity in a population of grasshopper sparrows (*Ammodramus savannarum* ) in Maryland. Wilson J. Ornithol. 130(1): 40-51.

[10]. Diniz, P., Macedo, R. H. & Webster, M. S. 2019. Duetting correlates with territory quality and reproductive success in a suboscine bird with low extra-pair paternity. Auk 136(1):1-13.

[11]. Eastwood, J. R., Berg, M. L., Ribot, R. F. H., Stokes, H. S., Martens, J. M. & Buchanan, K. L., et al. 2018. Pair fidelity in long-lived parrots: genetic and behavioural evidence from the crimson rosella (*Platycercus elegans*). Emu 118: 369-374.

For Review Only
